# Editorial: Novel Approaches to Improve Detection, Differentiation and Treatment in Mood Disorders

**DOI:** 10.3389/fpsyt.2022.837283

**Published:** 2022-03-04

**Authors:** Karim Abdel Aziz, Andrés Herane-Vives, Emmanuel Stip, Danilo Arnone

**Affiliations:** ^1^Department of Psychiatry and Behavioural Science, College of Medicine and Health Sciences, United Arab Emirates University, Al Ain, United Arab Emirates; ^2^Institute of Cognitive Neuroscience, University College London, London, United Kingdom; ^3^Centre for Affective Disorders, Department of Psychological Medicine, Institute of Psychiatry, Psychology, and Neuroscience, King's College London, London, United Kingdom; ^4^Center for Integrative Biology, Universidad Mayor, Providencia, Santiago, Chile; ^5^Institute Universitaire en Santé Mentale de Montréal, Université de Montréal, Montréal, QC, Canada

**Keywords:** mood disorders, major depressive disorder, clinical differentiation, treatment, bipolar disorder

Mood disorders are a group of common conditions with uncertain disease mechanisms and unsatisfactory treatment outcomes which significantly affect quality of life (1). Refining our understanding of the aetiology of these conditions and improving treatment response are essential next steps to improve outcome. This topic includes research which aims at detection, characterisation and differentiation within mood disorders, with a particular focus on aetiological mechanisms and pathways to improve treatment response.

Eight articles published in this topic originated from China (approximately 70%), one from Taiwan, one from South Korea and one from Europe/North America. We hope that the studies presented in this topic contribute to the quest of exploring novel approached to increase precision in the identification and treatment of mood disorders.

Cognitive changes in mood disorders are becoming increasingly important in relation to mechanisms of disease onset and progression and as potential targets for personalised interventions (2–4). In this topic Ronold et al. assessed cognitive function and depressive symptoms in unipolar major depression 5 years after the first diagnosis. Authors identified persistence of abnormalities in executive functions that might be independent of depressive symptoms. In synergy, Chen W-Y. et al. investigated the cognitive function of patients with bipolar disorder type I. In their retrospective analysis of around 6.5 years, the authors found that bulk of episodes with psychotic features was an independent risk factor for cognitive decline with a steeper decline after age of 42. These studies taken together show that cognitive dysfunction within mood disorders might differ in trajectory requiring a diversified approach to optimise prevention and treatment.

In relation to the importance of early detection and preventative approaches, Chen X. et al. describe MoodBox, an internet-based psychological intervention designed to improve the psychological well-being of individuals experiencing subclinical depressive symptoms aiming at reducing transition into major depression. The intervention, to be tested in a multicentre, randomised, non-blinded, superiority study intends to compare MoodBox with an online psychoeducation programme and a naturalistic observational group for 8 weeks with a further 1 year follow-up phase.

There is interest in identifying potential novel therapeutic targets in major depression. Psilocybin is a novel approach currently under investigation for treatment refractory major depression which benefits from psychological assistance (5). The training that therapists receive to support this intervention is important. Tai et al. describe a new manualized evidence-based psychotherapeutic approach to assist psilocybin therapy. The intervention, developed in partnership with different mental health researchers, practitioners and experts across the US, Canada and Europe is approved by the US Food and Drug Administration. Li et al. describe the role of peripheral facial injection of botulinum neurotoxin type A in major depression. Preclinical models suggest that anti-depressant effects might be associated with up-regulation of serotonin levels and expression of brain-derived neurotrophic factor in the hippocampus. Authors conclude that with limitations, clinical data are encouraging and suggest that this therapy is a potential effective and safe intervention for the management of depression. Song et al. investigated antithrombin III (ATIII) as a potential biomarker for the evaluation and prediction of treatment response in major depression. The authors randomised patients to treatment with occipital repetitive transcranial magnetic stimulation (rTMS) or sham treatment for 5 days. The results revealed a reduction in ATIII after occipital rTMS in depressed patients and a relationship between change in ATIII and therapeutic response to occipital rTMS.

Studies evaluating differentiation within mood disorders and between mood disorders and other mental health conditions are particularly important to improve disease specificity at the point of diagnosis and treatment. Ge et al. described a network analysis method to map the presence of anxiety symptoms in individuals with major depression to potentially increase diagnostic precision and target interventions to reduce the occurrence of treatment resistance. Results revealed eight co-occurring symptoms in the network structure. Dong et al. investigated differences in gut microbiota between representative cases of major depressive disorder and general anxiety disorder. The study elucidated a gut-microbiome signature associated with these two conditions that might facilitate differential diagnosis and targeted therapeutic interventions.

In relation to developing cost effective approaches which provide innovative and simple solutions for diagnosis or help to identify those in need of supportive measure or predictors of treatment response, there are several new technologies which have recently been adopted in mental health including computer-based applications (6). Lee et al. provide a literature review of the use of wearable devices and sensors in patients with depression and discuss issues regarding utility, reliability, users' perspectives and privacy. Wang et al. investigated the association between depression and gait characteristics with the aim to assist the diagnosis of depression by using support vector machine algorithms. The most efficient model they described used time- and frequency-domain features with a very high specificity, suggesting that depression could be effectively recognized through gait analysis.

Finally, the article by Qian et al. investigated the role of chromatin remodelling which included histone acetylation in an animal model of major depression. The authors showed an association between depressive-like behaviours induced by chronic social defeat stress in mice and a decrease in the class II histone deacetylase HDAC7 in the nucleus accumbens. The works suggests that HDAC7 might be a promising therapeutic target for depression.

The work published in this issue adds to recent discoveries in mood disorders and contribute to improve aetiological and detection pathways which offer new opportunities for developing novel treatments (7). Some of the most interesting recent advances in mood disorders include the realisation that epigenetic markers can transmit across generations (8), the validation and consistent replication of structural and functional neuroimaging changes in mood disorders (9–11), new developments in the techniques to analyse co-morbidities at brain level (12), the development of multi-essays serological tests to integrate biological markers with clinical phenotypes (13). Esketamine and more recently Lumateperone are among the most novel approved new treatments for mood disorders (14, 15), whereas esmethadone (16) and new neuromodulation techniques such as transcranial alternating and direct current stimulation (17, 18) are some of the new approaches under consideration.

In conclusion although mood disorders are complex condition which offer considerable challenges at different levels from the recruitment of research participants (19) to limited knowledge at brain level (20), our understanding of aberrant processes is expanding and new treatments are increreasingly becoming available.

## Author Contributions

KA, AH-V, and ES contributed to writing of the first and subsequent drafts. DA conceived the idea, contributed to the writing of the manuscript, and supervised the work. All the authors approved the final version of the manuscript.

## Conflict of Interest

The authors declare that the research was conducted in the absence of any commercial or financial relationships that could be construed as a potential conflict of interest.

## Publisher's Note

All claims expressed in this article are solely those of the authors and do not necessarily represent those of their affiliated organizations, or those of the publisher, the editors and the reviewers. Any product that may be evaluated in this article, or claim that may be made by its manufacturer, is not guaranteed or endorsed by the publisher.
